# The burden of persistent symptom diagnoses in primary care patients: a cross-sectional study

**DOI:** 10.1080/02813432.2023.2293930

**Published:** 2024-02-07

**Authors:** Asma Chaabouni, Juul Houwen, Georg Grewer, Martin Liebau, Reinier Akkermans, Kees van Boven, Iris Walraven, Henk Schers, Tim Olde Hartman

**Affiliations:** a Department of Primary and Community care, Radboud Institute of Health Sciences, Radboud University Medical Centre, Nijmegen, The Netherlands; b USUMA Markt- und Sozialforschung GmbH, Berlin, Germany; c Scientific Institute for Quality of Healthcare, Radboud Institute of Health Sciences, Radboud University Medical Centre, Nijmegen, The Netherlands; d Department for Health Evidence, Radboud Institute of Health Sciences, Radboud University Medical Centre, Nijmegen, The Netherlands

**Keywords:** primary care, persistent somatic symptoms, quality of life, disease burden

## Abstract

**Introduction:**

The burden of symptoms is a subjective experience of distress. Little is known on the burden of feeling unwell in patients with persistent symptom diagnoses. The aim of this study was to assess the burden in primary care patients with persistent symptom diagnoses compared to other primary care patients.

**Methods:**

A cross-sectional study was performed in which an online survey was sent to random samples of 889 patients with persistent symptom diagnoses (>1 year) and 443 other primary care patients after a transactional identification in a Dutch primary care data registry. Validated questionnaires were used to assess the severity of symptoms (PHQ-15), Symptom Intensity and Symptom Interference questionnaires, depression (PHQ-9), anxiety (GAD-7), quality of life (SF-12 and EQ-5D-5L)) and social functioning (SPF-ILs).

**Results:**

Overall, 243 patients completed the survey: 178 (73.3%) patients in the persistent symptom diagnoses group and 65 (26.7%) patients in the control group. In the persistent group, 65 (36.5%) patients did not have persistent symptom(s) anymore according to the survey response. Patients who still had persistent symptom diagnoses (*n* = 113, 63.5%) reported significantly more severe somatic symptoms (mean difference = 3.6, [95% CI: 0.24, 4.41]), depression (mean difference = 3.0 [95% CI: 1.24, 3.61]) and anxiety (mean difference = 2.3 [95% CI: 0.28, 3.10]) and significantly lower physical functioning (mean difference = − 6.8 [95% CI: −8.96, −3.92]).

**Conclusion:**

Patients with persistent symptom diagnoses suffer from high levels of symptoms burden. The burden in patient with persistent symptoms should not be underestimated as awareness of this burden may enhance person-centered care.

## Introduction

General practitioners (GPs) have become increasingly aware of the importance to assess and monitor the burden of health problems as part of the emphasis on person-centered approach in primary care, a perspective that requires to understand the patient as a unique human being including focusing on the elements of care, support and treatment that matter most to the patient, their family and carers [1]. The burden of symptoms is defined as the subjective experience of distress related to health problems including the symptoms severity, patients’ daily functioning, psychological and social factors [2–6]. The level of burden is important to assess as it influences the course of a health problem, and ultimately its persistence. A growing body of research provides evidence that a high level of burden in patients with chronic conditions, such as diabetes and hypertension, negatively affects their wellbeing [7–9]. Therefore, awareness of the level of burden should be targeted to improve quality of care including person-centered approach.

Symptom diagnoses are defined as a wide range of symptoms when the relevant diagnostic criteria of a disease are not (yet) fulfilled [10–12]. Based on the ecology in primary care, symptom diagnoses are highly prevalent and constitute more than half of the total number of primary care diagnoses [12]. One in six patients have more than one contact with the GP for symptoms that persist for more than one year [12]. Most primary care guidelines on management of persistent symptoms target symptoms burden, more specifically symptoms severity as an indicator for a stepped care approach [3]. A stepped care approach is a model of health care delivery that provides regular monitoring, and according to the patient’s needs more intensive treatments [13]. Although such a stepped care approach showed positive effects on the symptom burden [14–16], GPs often prescribe unnecessary medication and referrals to specialists [17]. Indeed, GPs often assume that the level of burden is less severe in patients with persistent symptom diagnoses compared to patients with well-established conditions [18]. While most of the literature on the level of burden in primary care patients is focused on well-established conditions [7–9], little is known on the level of burden in patients with persistent symptom diagnoses. Knowledge on the level of burden is needed as these patients frequently request care from their GPs, yet often feel misunderstood and not taken seriously [18]. A better GPs’ understanding of the level of burden in these patients might enhance a person-centered approach. Therefore, this study aims to assess the level of burden in primary care patients with persistent symptom diagnoses compared to other primary care patients.

## Methods

We performed an observational cross-sectional study to explore the level of burden in patients with persistent symptom diagnoses and compared these levels to other primary care patients. All participants were identified and selected from the Family Medicine Network (FaMe-Net) database. The FaMe-Net database is a Practice Based Research Network in the Netherlands (https://www.famenet.nl) including 6 GP practices, 30 GPs and more than 40,000 enlisted patients in 2022. The FaMe-Net GPs code all doctor-patient contacts within an Episode of Care (EoC) structure. An EoC is defined as ‘a health problem in an individual from the first until the last encounter’ [19]. An EoC includes (1) the reason for encounter (RFE = the literal expression of the reason(s) why a person enters the consultation room) [20], (2) the diagnoses, (3) the interventions (diagnostic interventions, treatment, and referrals), and (4) all encounters within this EoC [19]. The RFE, diagnoses and interventions are coded following the 2^nd^ edition of the International Classification of Primary Care (ICPC-2) [21].

### Pre-registration and network

This study was pre-registered (DOI: 10.17605/OSF.IO/VT6P7), and has been performed according to open-science principles. The current study is part of the innovative training network ETUDE (Encompassing Training in fUnctional Disorders across Europe; https://etude-itn.eu/), a network that aims to improve the understanding of mechanisms, diagnosis, treatment and stigmatization of functional disorders [22].

### Participants

For patients with persistent symptom diagnoses, we selected all patients who had at least one contact with their GP between 01 July 2020 and 01 July 2022 for at least one symptom diagnosis that lasted for at least one year. A duration of an EoC was calculated as the difference between the first contact and the last contact within the study period. A contact with the GP could include face-to-face encounters, encounters during out-of-service hours, telephonic consultations, and e-consultations. The threshold of a duration of 1 year for persistent symptom diagnoses was determined based on the distribution of symptom duration in the FaMe-Net database combined with the opinions of practicing GPs experts in the field of symptom research [12]. An overview of all included ICPC-2 codes for symptom diagnoses, and the top 10 included ICPC-2 codes for symptom diagnoses in the persistent group in this study are summarized in the Appendices 1 and 2.

For the control group (i.e. other primary care patients), we selected all patients from the practice list other than patients as identified with a persistent symptom diagnoses.

We randomly selected patients from both identified groups stratified by the number of patients in the practice list of each GP practice. Because every citizen in the Netherlands is registered to a General Practice, the control group, namely all other primary care patients, reflects the general population as the control group was randomly selected from the patients practice lists. Selected patients in both groups were sent an online consent form by email. When patients consented to participate, they received an online survey. We sent two reminder emails to patients who didn’t complete the online survey within 2 to 4 weeks from the consent date. Patients under 18 years, who had an incorrect email address, left the practice or died, were excluded from the study. Patients initially allocated to the persistent group and who did not have the symptoms anymore when they completed the survey were allocated to a residual group and excluded from the analysis.

### Questionnaires

The survey included questions on personal characteristics (age, sex, level of education, marital status, employment, tobacco consumption, alcohol consumption and drugs use). Patients with a persistent symptom diagnosis, for which they contacted their GP over the last 2 years, were asked whether they still had the specific symptom as indicated in the FaMe-Net data registry. Besides personal characteristics, the survey included the following questionnaires: Patient-Health Questionnaire-15 (PHQ-15) [23], Symptom Intensity and Symptom Interference with daily activity questionnaire [24], Patient-Health-Questionnaire-9 (PHQ-9) [25], Generalized Anxiety Disorder 7 (GAD-7) [26], Short form health survey 12 (SF-12) [27], EuroQol Five Dimensional Five Levels Questionnaire (EQ-5D-5L) [28], and Social Production Functioning Scale-Short (SPF-ILs) [29]. These questionnaires were selected based on their validity and previous use in primary care and somatic symptoms populations [24]. Together, these questionnaires explored the main biopsychosocial core domains to assess the distress related to the symptoms, and therefore the symptoms burden [24, 30]

#### Primary outcome

*The Patient-Health Questionnaire-15 (PHQ-15)* is a 15-item questionnaire that measures the severity of somatic symptoms [23]. Participants were asked to answer the following question ‘‘Over the last 2 weeks, how often have you been bothered by any of the following problems?’’. Participants were asked to rate their symptoms based on a 3-point Likert scale ranging from 0 (not bothered at all) to 2 (bothered a lot). One item ‘‘Menstrual cramps or other problems with your period’’ was only asked to female participants. The overall score could range between 0 to 30 for females and 0 to 28 for males. An overall score of 10 or more represented moderate to severe symptoms.

#### Secondary outcomes

*Symptom Intensity and Symptom Interference with daily activity* are two numeric scales to assess the overall intensity of bodily symptoms and the overall symptom interference in daily life activities over the last 7 days [24]. Each scale is scored using an 11-point Likert scale ranging from 0 (no symptoms/not at all) to 10 (worst possible symptoms/interfered completely). These two self-administered scales are validated in English. The two scales were first translated by a native Dutch speaker fluent in English (JH) then a native English speaker fluent in Dutch (BMcG) was asked to translate the Dutch version into English without access to the original English version. A high level of similarities was found between the original English and the Dutch versions (Appendix 3). Only patients who still had persistent symptom diagnoses were asked to answer the symptom intensity and symptom interference with daily activities questionnaires. They were asked to refer back to their symptom(s) as indicated to the FaMe-Net database when answering these questions.

*The Patient Health Questionnaire-9 (PHQ-9)* is a 9-item questionnaire to measure depressive signs and symptoms over the last two weeks [31]. Each item is answered according to a 4-point Likert scale ranging from 0 (not at all) to 3 (nearly every day). The overall scores could range between 0 and 27. An overall score of 10 or more represented moderate to severe symptoms.

*The Generalized Anxiety Disorder 7-item (GAD 7)* is a 7-item measure to identify the frequency and severity of anxiety symptoms over the last two weeks [32]. Each item is answered according to a 4-point Likert scale ranging from 0 (not at all) to 3 (nearly every day). The overall score could range between 0 and 21. An overall score of 10 or more represented moderate to severe symptoms.

*Short form health survey 12 (SF-12)* is a questionnaire to measure subjective mental and physical health related quality of life [33]. The SF-12 includes 12 items categorized in eight dimensions: physical functioning, role limitations due to physical functioning (role functioning—physical), bodily pain, general health perception, vitality, social functioning, role limitations due to emotional functioning (role functioning—emotional), and general mental health. The time frame vary between ‘‘now’’ for the physical functioning dimension, and ‘‘during the past four weeks’’ for the other dimensions. The raw scores of each item are coded, weighted, and summed into two scales: physical component summary score (PCS) and mental component summary score (MCS). Higher scores indicate better quality of life [33].

*EuroQol Five-Dimensional Five Levels Questionnaire (EQ 5D-5L)* is a questionnaire to assess health related quality of life [34]. The EQ-5D-5L includes five domains including mobility, self-care, usual activities, pain/discomfort, anxiety/depression, with five point Likert scale responses that range from 1 (no problems) to 5 (extremely severe problems). For each domain, moderate to extremely severe problems starts from an overall score of 3. EQ-5D-5L also included an additional question, the Visual Analogue Score (VAS), to assess the general health status based on a numeric scale ranging from 0 (worst imaginable health status) to 100 (best imaginable health status). The time frame for the five domains and the numeric scale is ‘‘today’’. The EQ 5D-5L results were converted to health utility scores using the Dutch Tariff [28]. Utility scores ranged between −1 and 1 where higher scores represent a better health status.

*Social Production Functioning Scale-Short (SPF-ILs)* is a 15-item questionnaire to measure the five following domains: affection, status, behavioural confirmation, comfort and stimulation [29]. The time frame for the SPFI-ILs is ‘‘the past 3 months’’. The questions are answered on a 5-point Likert scale ranging from 0 (never) to 4 (always). An overall score could range between 0 and 60, with higher scores indicating greater social wellbeing [29].

### Sample size calculation

The estimated sample size was calculated based on the expected difference in prevalence of moderate to severe symptoms between the two groups. In previous studies, the prevalence of moderate to severe somatic symptoms using PHQ-15 scores (i.e. PHQ-15 ≥ 10) were found to be 14.4% for the control group [35–38] and 40.2% for the persistent symptoms group [38, 39]. Assuming a power of 80%, an alpha of 0,05 for two-sided testing, a total of 92 participants was required, 46 participants in each group (i.e. control and persistent symptom diagnoses groups). On the basis of previous research within this population [40], we expected a participation rate of 30%, a general drop-out rate of 60%, and a drop-out rate of 70% for participants assigned to the persistent symptom diagnoses group due to not experiencing the symptoms anymore. Hence, we selected 975 patients in the persistent symptom diagnoses group and 525 participants in the control group.

### Statistical analyses

Descriptive statistics were reported to summarize personal characteristics, primary and secondary outcome variables. Mean (Standard Deviation [SD]) or median (interquartile [IQR]) for continuous characteristics and number and frequencies (%) for categorical characteristics. To compare personal characteristics between patients with persistent symptom diagnoses and patients in the control group, Student’s independent sample *T*-test (for continuous variables) and a Pearson’s chi-squared test (for categorical variables) were performed. To study differences in the primary and secondary outcomes between patients with persistent symptom diagnoses and patients in the control group, linear regression for continuous variables and logistic regression for dichotomized variables were performed. Both *p* values and the effect size or odds ratio with 95% Confidence Intervals (CI) were reported. We controlled for confounders from the personal characteristics variables when significant differences were found between the persistent and the control groups. We used Cohen’s effect size to calculate the clinical relevance of the continuous outcome variables [41]. Cohen’s score equal or higher than 0.5 was considered as a clinically relevant difference [24]. Data were analyzed using Statistical Package for Social Science (SPSS) version 25.0 for Windows. A *p*-value of < 0.05, based on two-sided testing, was considered statistically significant.

## Results

### Recruitment and patient characteristics

**Figure 1.**
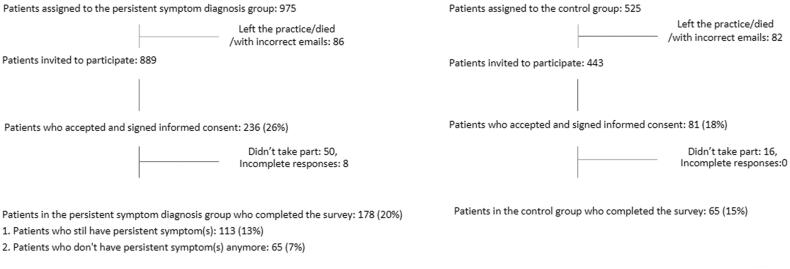
Flow chart of the recruitment process.

The consent form was sent to 1332 participants. Of them, 317 (23.8%) consented to participate: 236 patients in the persistent symptom diagnoses group and 81 patients in the control group. Overall, 243 patients completed the survey. Of them, 178 (73.2%) patients in the persistent symptom diagnoses group and 65 (26.7%) patients in the control group. From the 178 in the persistent group, 65 patients (36.5%) did not experience the persistent symptom(s) anymore (i.e. the residual group) (Figure 1). Therefore, we included 113 patients with a persistent symptom diagnosis in the analysis.

Patients in the persistent and control groups who completed the survey were significantly older (mean age = 56 years vs 49 years, *p* = 0.002) than patients who didn’t participate in the study. No significant difference was found in sex (males who participated: 67 (38%) versus males who didn’t participate: 535 (40%), *p* = 0.470).

Patients with persistent symptom diagnoses had a significantly lower level of education (primary/secondary school only: 27% versus 54%, *p* < 0.001), were significantly less likely to be employed (49% versus 65%, *p* = 0.040), reported more tobacco use (18% versus 4%, *p* = 0.011) and less alcohol use (60% versus 77%, *p* = 0.02) compared to other primary care patients (i.e. control group) (Table 1).

**Table 1.** Patients characteristics in the persistent and the control groups.

	Persistent symptoms group (*n* = 113)	Control group (*n* = 65)	sig
Mean age in years (SD)	58 (15)	54 (17)	0.150
Sex (Males)	37 (33%)	30 (46%)	0.075
Marital status			0.787
Single	37 (33%)	21 (32%)	
In a relationship	16 (14%)	7 (11%)	
Married	60 (53%)	37 (57%)	
Level of education			**<0.001**
Primary/secondary school	61 (54%)	18 (27%)	
University	52 (46%)	47 (73%)	
Employment (Yes)	55 (49%)	42 (65%)	**0.040**
Tobacco consumption (Yes)	20 (18%)	3 (4%)	**0.011**
Alcohol consumption (Yes)	68 (60%)	50 (77%)	**0.023**
Drugs use (Yes)	1 (1%)	1 (2%)	1.000

Chi square was used with the exception of Age where independent sample *t*-test was used.

Regarding the control group, 3 (4.6%) patients didn’t have any contact with the GP between July 2020 and July 2022. The median number of contacts of patients in the control group was 1 (IQR = 1 – 2), and the median number of EoC was 5 (IQR= 3.75 − 9.25). The most frequent diagnoses were ‘‘Health maintenance/prevention’’ (ICPC-2: A98) and ‘‘Bursitis/tendinitis/synovitis’’ (ICPC-2: L87). The top 10 diagnoses of participants in the persistent symptom and the control groups are detailed in Appendix 2.

### Level of burden

#### Primary outcome

After controlling for the level of education, employment status, tobacco and alcohol use, patients with persistent symptom diagnoses reported significantly more severe somatic symptoms compared to patients in the control group (mean difference in PHQ-15 scores = 3.6, [95% CI: 0.24,4.41], *p* < 0.001) (Tables 2 and 3). Cohen’s size effect was 0.9 reflecting a clinically relevant difference. Forty-eight (42%) patients in the persistent symptom group reported a score of 10 or higher indicating moderate to severe somatic symptoms, compared to 7 (11%) patients in the control group (OR = 4.91 [95% CI: 1.97,12.20], *p* < 0.001). The PHQ-15 showed a good level of internal consistency in our study (Cronbach’s α was 0.79).

**Table 2.** Primary and Secondary outcome variables in the persistent and control groups.

	Persistent symptoms group (*n* = 113)	Control group (*n* = 65)
*Primary outcome*		
PHQ-15 (Mean [SD])	8.8 [5.3]	5. 2 [3.3]
*Secondary outcomes*		
Symtom intesity (Median [IQR])	6 [4 − 7]	–
Symptom interference (Median [IQR])	5 [3 − 7]	
PHQ-9 (Mean [SD])	6.9 [5.7]	3.9 [4.5]
GAD-7 (Mean [SD])	4.9 [4.9]	2.6 [2.9]
SF-12		
PCS (Mean [SD])	36.4 [8.2]	43.2 [6.8]
MCS (Mean [SD])	37.4 [6.3]	37.0 [4.8]
EQ 5D 5 L		
Mobility problems (n, %)	67 (59.0%)	23 (35.0%)
Self-care problems (n, %)	20 (17.7%)	7 (10.6%)
Usual activities problems (n, %)	64 (57.0%)	17 (26.0%)
Pain (n, %)	94 (83.0%)	40 (62.0%)
Anxiety/depression levels (n, %)	49 (43.0%)	21 (32.0%)
Visual Analogue Score for general health status (Mean [SD])	64.9 [22.6]	76.4 [15.8]
Utility score (Mean, [SD])	0.61 [0.3]	0.80 [0.2]
Social Production Function Instrument		
Overall (Mean [SD])	39.6 [5.8]	42.0 [6.6]
Affection (Mean [SD])	9.1 [9.0]	9.4 [1.9]
Behavioural Confirmation (Mean [SD])	9.3 [9.0]	9.5 [1.9]
Status (Mean [SD])	6.5 [6.0]	6.8 [1.9]
Comfort (Mean [SD])	6.6 [7.0]	8.1 [1.9]
Stimulation (Mean [SD])	8.0 [8.0]	8.1 [1.4]

PHQ-15: Patient Health Questionnaire-15, higher scores reflect more severe somatic symptoms; PHQ-9: Patient Health Questionnaire-9, higher scores reflect higher level of depression; GAD-7: Generalized Anxiety Disorder-7, higher scores reflect higher levels of anxiety; SF-12: Short Form-12, Higher score reflect better health related quality of life, PCS = Physical Component Summary Score; MCS = Mental Component Summary Score; EQ 5D 5 L: Five Dimensional Five Levels Questionnaire, The proportion reported for each domain for the EQ-5D-5L are those that reported a score of 3 or greater which represents a moderate to extremely severe problem, Affection = positive inputs from caring others, Behavioural Confirmation = approval for doing the ‘right things’. Status = control over scare resources, Comfort = absence of physiological needs, pleasant and safe environment, Stimulation = Activation optimal level of arousal.

**Table 3.** Severity of symptoms, depression, anxiety, social wellbeing and quality of life levels between the persistent and the control groups.

	Crude analysis		Adjusted analysis^a^		
Outcomes	95% CI^b^	*p* values	95% CI^b^	*p* values	Cohen’s size effect
*Primary outcome*					
PHQ-15	**3.55, [2.12, 4.98]**	**<0.001**	**2.91, [1.41, 4.41]**	**<0.001**	**0.9**
*Secondary outcomes*					
PHQ-9	**2.97, [1.34, 4.58]**	**<0.001**	**1.92, [0.24, 3.61]**	**0.026**	**0.4**
GAD-7	**2.32, [0.98, 3.67]**	**<0.001**	**1.69, [0.28, 3.10]**	**0.019**	**0.6**
SF-12					
PCS	**−6.78, [−9.16, −4.41]**	**<0.001**	**−6.44, [−8.96, −3.92]**	**0.001**	**1.0**
MCS	0.41, [−1.37, −2.19]	0.649	−0.02, [**−**1.87, 1.90]	0.987	–
EuroQual 5D					
Mobility problems	**2.66, [1.41, 5.00]**	**0.002**	**2.27, [1.14, 4.52]**	**0.020**	**-**
Self-care problems	1.78, [0.71, 4.48]	0.219	1.20, [0.43, 3.34]	0.734	–
Usual activities problems	**3.69, [1.99, 7.18]**	**0.001**	**2.95, [1.46, 5.93]**	**0.002**	**-**
Pain	**3.09,[1.53, 6.24]**	**0.002**	**2.76, [1.29, 5.88]**	**0.009**	**-**
Anxiety/depression levels	1.60, [0.85, 3.04]	0.147	1.25, [0.62, 2.50]	0.530	–
The Visual Analogue Score for general health status	**−11.56, [−17.82, −5.31]**	**< 0.001**	**−8.39, [−14.95, −1.83]**	**0.012**	**0.5**
Utility score	**−0.18, [−0.27, −0.10]**	**< 0.001**	**−0.13, [−0.21, −0.05]**	**0.003**	**0.8**
Social Production Function Instrument					
Overall score	**−2.32,[−4.19, −0.45]**	**0.015**	−1.33, [**−**3.28, 0.63]	0.182	**-**
Affection	−0.28, [**−**0.86, 0.30]	0.337	−0.20, [**−**0.82, 0.42]	0.525	–
Behavioural Confirmation	−0.16, [**−**0.70, 0.37]	0.550	−0.09, [**−**0.64, 0.47]	0.760	–
Status	−0.32, [**−**0.92, 0.28]	0.291	−0.02, [**−**0.65, 0.61]	0.949	–
Comfort	**−1.44, [−2.07, −0.80]**	**<0.001**	**−1.08, [−1.74, −0.42]**	**0.001**	**0.5**
Stimulation	−0.12, [**−**0.59, 0.35]	0.568	−0.06, [**−**0.43, 0.55]	0.807	–

Linear regression for continuous variables and logistic regression for dichotomized variables; Cohen’s size effect was not calculated for categorical variables nor for after non- significant different after adjusting for confounders;

^a^ Adjusted for level of education, employment, tobacco and alcohol consumption;

^b^ estimates were reported for continuous outcome variables and odds ratios were reported for categorical outcomes = variables; PHQ-15: Patient Health Questionnaire-15, higher scores reflect more severe somatic symptoms; PHQ-9: Patient Health Questionnaire-9, higher scores reflect higher level of depression; GAD-7: Generalized Anxiety Disorder-7, higher scores reflect higher levels of anxiety; SF-12: Short Form-12, Higher score reflect better health related quality of life, PCS = Physical Component Summary Score; MCS = Mental Component Summary Score; EQ 5D 5 L: Five Dimensional Five Levels Questionnaire, The proportion reported for each domain for the EQ-5D-5L are those that reported a score of 3 or greater which represents a moderate to extremely severe problem, Affection = positive inputs from caring others, Behavioural Confirmation = approval for doing the ‘right things’. Status = control over scare resources, Comfort = absence of physiological needs, pleasant and safe environment, Stimulation = Activation optimal level of arousal.

#### Secondary outcomes

Patients in the persistent symptom diagnoses group reported significantly higher scores for depression (mean difference in PHQ-9 score = 3.0 [95% CI: 1.24, 3.61], *p* = 0.026) and anxiety (mean difference in GAD-7 score = 2.3 [95% CI: 95 0.28, 3.10], *p* = 0.019) compared to patients in the control group. Cohen’s size effect for depression and anxiety were 0.4 and 0.6 respectively. PHQ-9 and GAD-7 showed good levels of internal consistency (Cronbach’s α of 0.88 and 0.92 respectively). Twenty-four patients (21%) in the persistent symptom group reported a score above clinical threshold of 10 or higher in PHQ-9 indicating moderate to severe depressive symptoms, compared to 7 (11%) in the control group (OR = 0.70, 95% CI [0.09, 5.49], *p* = 0.730). Seventeen (15%) of patients in the persistent symptom group reported a score above clinical threshold of 10 or higher in GAD-7 indicating moderate to severe anxiety symptoms, compared to 1 (1%) in the control group (OR = 8.35, 95% CI [1.03, 67.44], *p* = 0.046).

A lower physical functioning was found in the persistent symptom diagnoses group compared to the control group (mean difference in PCS score = − 6.8 [95% CI: −8.96, −3.92], *p* < 0.001) with a clinical relevant difference (Cohen’s effect size = 1). Patients with persistent symptoms reported significantly more mobility problems (59% vs 35%, OR = 2.27, [95% CI: 1.14, 4.52], *p* = 0.020), usual activities problems (57% vs 26%, OR = 2.95, [95% CI: 1.46, 5.93], *p* = 0.002) and pain (83% vs 62%, OR = 2.76, [95% CI: 1.29, 5.88], *p* = 0.009] than patients in the control group.

When quality of life was summarized in utility scores, the utility score was significantly lower in patients with persistent symptom diagnoses compared to patients in the control group (mean difference = − 0.13, [95% CI: −0.21, −0.05], *p* = 0.003) with a clinical relevant difference (Cohen’s effect size = 0.8).

No statistically significant differences were found for patients with persistent symptom diagnoses as compared to patients in the control group for social well-being and the remaining domains of health-related quality of life including the mental component score (MCS), mental health problems, and self-care problems (Tables 2 and 3).

## Discussion

### Summary of the main findings

In this cross-sectional study, we assessed the level of burden in primary care patients with persistent symptom diagnoses. Around one third of the primary care patients with persistent symptom diagnoses didn’t have the symptom(s) anymore when completing the survey. However, when the symptoms were still persistent, patients reported significantly higher levels of severity in somatic symptoms compared to other primary care patients. Additionally, patients with persistent symptom diagnoses reported higher levels of depression and anxiety. Notably, general utility and the physical functioning were significantly lower in patients with persistent symptom diagnoses. Mental functioning and social wellbeing did not significantly differ in patients with persistent symptom diagnoses and other primary care patients.

### Comparison to the literature

To our knowledge, this is the first study to explore the level of burden in primary care patients with persistent symptom diagnoses. Previous literature on the level of burden in patients with persistent somatic symptoms has explored populations with different diagnostic criteria such as Medically Unexplained Symptoms (MUS) or somatoform disorders [35–38]. The latter diagnostic labels have been criticized for being imprecisely defined as they both require the concept of ‘‘explanation of the symptoms’’ which reduces the symptoms as either being ‘physical’ or ‘non-physical’ [10]. In this study, we explored the level of burden among patients with a wide range of persistent complaints and using a diagnostic label (i.e. persistent symptom diagnoses) that is more relevant for GPs in clinical practice and less harmful for patients [10, 38, 39]. Patients with symptom diagnoses, MUS and somatoform disorders are partially overlapping groups. However, symptom diagnoses include all symptoms that the patient can present with during a primary care consultation. It has been preferred as an alternative in order to come to a more holistic approach to the practice of family medicine [10].

Previous literature on the level of burden in MUS and somatoform disorder have found comparable findings with our study. Indeed, primary care patients with persistent symptoms in these studies consistently reported increased levels of moderate to severe levels of somatic symptoms ranging between 40% and 50% [38, 39] compared to the general population where moderate to severe levels ranged between 9% and 15% [35–38]. Similarly, previous studies have reported increased psychological problems including depression and anxiety, physical functioning impairment, as well as decreased quality of life and general utility compared to other primary care patients [18, 42–45]. These similarities in increased burden in patients with persistent symptoms might suggest that illness consequences are overlapping between patients with persistent symptom diagnoses and patients with other diagnostic labels.

While anxiety and depression levels in the GAD-7 and PHQ-9 respectively, were significantly higher in patients with persistent symptom diagnoses compared to other primary care patients, no significant difference was found in the anxiety/depression domain using EQ-5D-5L. Indeed, the use of EQ-5D-5L increases the risk of missing out positive cases of depression and anxiety compared to other anxiety and depression self-report screening tools [46]

Patients with persistent symptom diagnoses in this study did not report lower mental functioning compared to other primary care patients. In previous literature, findings were conflicting. Some studies have found that physical functioning is predominantly affected in patients with persistent symptoms, while mental functioning shows comparable levels to patients with non-persistent symptoms [43, 47]. A Dutch study on persistent MUS [42], using the same measure (SF36) as the other studies [43, 47], found that mental functioning was also affected. This contradiction in findings might suggest that the influence of persistent symptoms on the perceived mental functioning is affected by several extra factors such as age and self-efficacy beliefs [48]. Additionally, other diagnostic labels used in previous studies have been described to be stigmatizing, and therefore might lead to consequences on quality of life including a decreased mental functioning [49].

Surprisingly, in this study, social wellbeing was not significantly more affected in patients with persistent symptoms than in other primary care patients. This finding suggests that having persistent symptom diagnoses does not affect social wellbeing, more specifically people’s social needs [29]. Previous studies on patients with persistent symptoms have demonstrated that other social elements playing a role in people’s social wellbeing, such as social isolation [42], social adjustment and difficulties in relationships [45], are more prevalent in patients with persistent symptoms. Therefore, even though social needs seem to be achieved in patients with persistent symptoms, other social elements might be affected.

Interestingly, 1 in 3 patients who had contacted their GP for persistent symptom diagnoses didn’t experience the symptom(s) anymore. This might be explained by the fluctuating course of persistent symptom diagnoses as previous literature has shown that the course of persistent symptoms is fluctuating in 80% of the cases [50]

### Strengths and limitations

This study has several strengths. First, we identified a large number of patients with and without persistent symptom diagnoses from the FaMe-Net dataset, a valid and structured primary care electronic health record database, that has been demonstrated to be representative for the Dutch population regarding age, sex and social class [19, 51]. We also included a sufficient number of patients with a large number of persistent symptom diagnoses, for which they contacted their GP. These primary care patients with persistent symptoms are known to represents 20% of the population who might experience symptoms [52]. This inclusion reflects the diversity of the population in primary care settings and improves the power of the statistical analyses.

Secondly, we asked patients with persistent symptoms to refer back to their specific symptom(s) as indicated in the FaMe-Net dataset when answering questions about their level of burden. This might decrease response bias that might be found in other studies in which patients with persistent symptoms were identified by self-administered questionnaires [18, 53] or new interviews [43, 54].

We acknowledge several limitations of this study. First, we have not studied specific-individual differences which might have interfered with the level of symptom burden, such as identity, sense making, a history of traumatic events, employment and subjective exploratory models [6, 55]. Even though we have made the choice of comparing the persistent symptom diagnoses group to other primary care patients, matching the control and the persistent symptom groups on the symptoms level might have decreased the gap between the burden of symptom differences in the compared groups. A second limitation could be that the burden level in patients with persistent symptoms might be overestimated in this study. For instance, as patients who were included in the study were significantly older than patients who did not participate, additional age-related health concerns might have increased the level of burden. Even though the number of participants needed in each group was higher than the minimum number as determined in the power calculation, a third limitation could be the low response rate which could lead to a selection bias.

### Future research and clinical implications

This study demonstrates that patients with persistent symptom diagnoses often suffer from higher levels of burden compared to other primary care patients. This finding may enhance GPs awareness of the level of burden in patients with persistent symptom diagnoses. Aligned with the person-centered approach, our findings suggest that when the symptoms persist, GPs should carefully assess the patient’s thoughts, feelings and social interactions, factors that are all rooted in the bio-psycho-social model [30]. Such a careful assessment may result in a better understanding of the patients’ illness experience and guide GPs in a stepped care approach [3]. For primary care patients with a high level of burden, several treatment options are recommended in guidelines such as psycho-education, psychosomatic therapy and cognitive behavioural therapy [3, 14, 16]. Future studies on the level of burden might focus on longitudinal diary monitoring as an approach to gather all relevant bio-psycho-social factors relevant for the individual, which might optimize the choice of a personal management strategy in patients with persistent symptom diagnoses. To provide further understanding, future qualitative studies might consider the assessment of the level of support, attitudes of the social network of patients with persistent symptom diagnoses, and other factors that helped in improving the symptoms in patients who don’t experience the symptoms anymore.

## Appendix 1: Overview of all studied symptom diagnoses from the international classification of primary care, 2^nd^ edition (ICPC-2)

**Table:**

Selected ICPC-2 codes: Chapters A to Y: All the codes from 1 to 29
Non-selected ICPC-2 codes: Risk factors and euthanasia Chapter A: A20 (Euthanasia Request/ decision), A21 (Risk factor for malignancy), A23 (Risk factor NOS) Chapter K: K22 (Risk factor cardiovascular disease) Diseases Chapter S: S03 (Warts), S09 (Infected finger/toe) S10 (Boil/carbuncle), S11 (Skin infection post-traumatic), S12 (Insect bite/sting), S13 (Animal/human bite), S14 (burn/scald), S15 (Foreign body in the skin), S16 (Bruise /contusion), S17 (Abrasion/scratch/blister), S18 (Laceration/cut), S19 (Skin injury/other), S20 (Corn/ Collosity) Family planning Chapter W: W10 (contraception postcoital), W11 (Contraception oral), W12 (Contraception Intrauterine), W13 (Sterilization), W14 (Contraception other) Chapter Y: Y13 (Sterilization male), Y14 (family planning male other) Psychological symptoms Chapter P: All codes from 1 to 29 Social problems Chapter Z: All codes from 1 to 29

## Appendix 2: Top 10 diagnoses in the persistent and the control groups

**Table:**

ICPC-2 codes in the persistent group (Label)	Frequencies (%) (*n* = 150)
A04 (Weakness/tirdness general)	8 (5%)
K04 (Palpitation/ awarness of heart)	8 (5%)
X11 (Menopausal symptom/ complaint)	8 (5%)
D12 (Constipation)	6 (4%)
L03 (Low back pain complaint)	6 (4%)
R05 (Cough)	6 (4%)
D06 (Abdominal pain localized other)	5 (3%)
L14 (Leg/thigh symptom/complaint)	5 (3%)
L15 Knee symptom/complaint	5 (3%)
R02 Shortness of breath/ dyspnea	5 (3%)
ICPC-2 codes in the control group (Label)	Frequencies (%) (*n* = 406)
A98 (Health maintenance/ prevention)	62 (15%)
L87 (Bursitis/ tendinitis/synovitis NOS)	13 (3%)
H81 (Excessive ear wx)	10 (2%)
S99 (Skin disease other)	8 (2%)
K86 (Hypertension uncomplicated)	6 (1%)
U71 (Cystitis/urinary infection other)	6 (1%)
L81 (Injury musculoskelatel NOS)	5 (1%)
P78 (Neuroaesthenia/ surmenage)	5 (1%)
S88 (Dermatitis contact/ allergic)	5 (1%)
A20 (Euthanasia request/ discussion)	4 (1%)

ICPC-2: International classification of primary care 2^nd^ edition, NOS: Not otherwise specified.

## Appendix 3: Symptom intensity and symptom interference items in Dutch

Gedurende de afgelopen 7 dagen was de algehele intensiteit van mijn lichamelijke symptomen: Helemaal geen symptomen [0], Ergst mogelijke symptomen [10].

Gedurende de laatste 7 dagen hebben mijn lichamelijke symptomen mijn dagelijkse activiteiten verstoord: Helemaal niet verstoord [0], Volledig verstoord [10].
